# Factors associated with long Covid in nursing professionals

**DOI:** 10.1590/1980-220X-REEUSP-2024-0268en

**Published:** 2025-01-31

**Authors:** Dayane de Souza Soares Vasconcelos, Daniele Alcalá Pompeo, Mayara Caroline Ribeiro Antonio-Viegas, Adriana Inocenti Miasso, Guilherme Oliveira de Arruda, Elen Ferraz Teston, Erlandson Ferreira Saraiva, Bianca Cristina Ciccone Giacon-Arruda

**Affiliations:** 1 Universidade Federal de Mato Grosso do Sul, Campo Grande, MS, Brasil.; 2 Faculdade de Medicina de São José do Rio Preto, São José do Rio Preto, SP, Brasil.; 3 Universidade Federal de Mato Grosso do Sul, Três Lagoas, MS, Brasil.; 4 Universidade de São Paulo, Escola de Enfermagem de Ribeirão Preto, Ribeirão Preto, SP, Brasil.

**Keywords:** Post-Acute COVID-19 Syndrome, Nurse Practitioners, Resilience, Psychological, Mental Health, Síndrome Post Agudo de COVID-19, Enfermeras Practicantes, Resiliencia Psicológica, Salud Mental

## Abstract

**Objective:**

To analyze the association between sociodemographic variables, lifestyle and mental health habits, and long Covid in nursing professionals.

**Method:**

Quantitative, observational, cross-sectional and analytical study, with 109 nursing professionals who had Covid-19 between 2020 and 2022. Data collection was carried out using an online form, with the following variables of interest: resilience, subjective well-being, age, sex, professional category, vaccination, physical activity, presence of symptoms resulting from Covid-19 infection after the acute phase of the disease and long Covid. Data were analyzed descriptively and inferentially using Poisson regression with robust variance and a significance level of 5%.

**Results:**

Male sex, high resilience, and high positive affect decrease the prevalence of long Covid by 71% (RP = 0.29), 40% (RP = 0.60) and 43% (RP = 0.57), respectively, while being in the nursing technician category increases its prevalence by 74% (RP = 1.74).

**Conclusion:**

The results emphasize the importance and support the development of promotion, prevention, treatment, and rehabilitation actions for individuals with long Covid. To achieve this, multidisciplinary care, centered on the person and directed at the context and work environment is required.

## RESUMEN

**Objetivo:**

Objetivo: Analizar la asociación entre variables sociodemográficas, hábitos de vida y salud mental y Síndrome Post Agudo de COVID-19 en profesionales de enfermería.

**Método:**

Estudio cuantitativo, observacional, transversal y analítico, con 109 profesionales de enfermería que tuvieron Covid-19 entre 2020 y 2022. La recogida de datos se realizó a través de un formulario online, con las variables de interés: resiliencia, bienestar subjetivo, edad, sexo, categoría profesional, vacunación, actividad física, presencia de síntomas derivados de la infección por Covid-19 tras la fase aguda de la enfermedad y Síndrome Post Agudo de COVID-19. Los datos se analizaron de forma descriptiva e inferencial mediante regresión de Poisson con varianza robusta y un nivel de significancia del 5%.

**Resultados:**

El género masculino, la alta resiliencia y el alto afecto positivo disminuyen la prevalencia de Covid largo en un 71% (RP = 0,29), 40% (RP = 0,60) y 43% (RP = 0,57), respectivamente, mientras que siendo de la categoría de técnico de enfermería. su prevalencia aumenta en un 74% (RP = 1,74).

**Conclusión:**

Conclusión: Los resultados refuerzan la importancia y apoyan el desarrollo de acciones de promoción, prevención, tratamiento y rehabilitación de personas con Síndrome Post Agudo de COVID-19. Para ello es necesaria una atención multidisciplinar, centrada en la persona y dirigida al contexto y entorno laboral.

## INTRODUCTION

From 2019 to 2023, the world was affected by the Covid-19 pandemic and experienced a status of health emergency worldwide^(1)^. In 2020, approximately 90,000 healthcare workers were infected in 30 countries^(1)^. In Brazil, as of June 2023, nursing professionals infected by the Covid-19 virus totaled 65,029, with 872 deaths, resulting in a fatality rate among the class of 2.27%^(2)^.

After the control and end of the pandemic, mainly enhanced by vaccination, studies have investigated a post viral infection health condition and described the different permanent changes in the physical and mental health of people who had the disease^(3,4)^, called long Covid^(5)^. This condition can appear up to three months after the acute phase of the infection, lasting at least two months^(5)^.

It is estimated that, worldwide, 200 million individuals have had prolonged symptoms following acute infection by the Covid-19 virus, with a prevalence of 43% of those who were infected^(6)^. In Brazil, a study carried out with 646 individuals^(7)^ showed that around 50.2% developed this condition. Although it can affect any person, women and healthcare workers appear to be at higher risk^(8)^.

Long Covid presents itself in different ways in individuals and may involve one or more symptoms, such as fatigue, loss of smell or taste senses, persistent cough, headache, muscle and joint pain, weakness, and malaise. In addition, impairment of psychomotor functions, neuropsychic changes such as concentration difficulties and memory loss or brain fog, dizziness, decline in language skills and verbal fluency are observed^(9)^.

These symptoms impact people’s ability to return to daily life, work, and social activities, which generates economic and mental health consequences for these individuals^(5,8,9)^. Available studies focus on investigating signs and symptoms of long Covid^(7)^, in the experience of the pandemic, and its effects on the health of professionals^(10)^, and the positive influence of the resilience shown by health professionals in coping with the symptoms caused by long Covid^(11)^. Therefore, it is urgent to investigate factors associated with long Covid, to contribute to the planning of strategic care actions for health professionals, especially with regard to lifestyle habits and mental health.

In view of the above, this study aimed to analyze the association of sociodemographic variables, lifestyle habits, and mental health with long Covid in nursing professionals.

## METHOD

### Design of Study

This is a quantitative, observational, cross-sectional, and analytical study. This study derives from a matrix study, with multivariates called “Long Covid in nursing professionals and its implications on mental health and functional status”. For its structure and organization, the recommendations of the declaration *Strengthening the Reporting of Observational Studies in Epidemiology* (STROBE)^(12)^ were followed.

### Population and Study Local

The study had national coverage, with nursing professionals (nursing technicians and nurses) living anywhere in Brazil. The inclusion criteria were: being a nursing professional, of both sexes, who reported having tested positive for Covid-19 through the RT-PCR test, from 2020 to 2022, regardless of the severity and treatment measures taken. Those who reported not having performed the Covid-19 test or whose test was not the RT-PCR were excluded.

The study sample was non-probabilistic, for convenience, and consisted of 109 nursing professionals (nursing technician and nurse).

### Variables of Interest and Data Collection Instruments

The variables of interest in this study were defined as: 1) response variable - long Covid; 2) explanatory variables – resilience levels, subjective well-being (positive and negative affects), age, sex, professional category, vaccination against Covid (yes/no), physical activity practice (yes/no), and presence of symptoms resulting from infection by Covid-19, after the acute phase of the disease.

For the variables age, sex (female/male), professional category (nurse/nursing technician), vaccination against Covid (yes/no), and physical activity practice (yes/no) an instrument was constructed by the study researchers that contained, in addition to these variables, others such as: municipality and state of residence, income, current place of work, and when tested positive for Covid, whether currently working in the area, medications in continuous use, and diagnosis of other health conditions.

The variables symptoms of Covid after acute infection and long Covid were assessed using a questionnaire, also developed by the researchers of this study, based on a previous study that analyzed the impacts of the pandemic on nurses’ health involving the main prolonged symptoms identified in the literature^(4)^: headache; muscle pain; weakness; cough; shortness of breath; memory problems/brain fog; sleep disturbances; and anxiety. For these symptoms, the participant was asked to indicate: 1) which ones persisted for at least four weeks after the acute phase; and 2) which symptoms remained/remain.

Furthermore, the questionnaire contained a question in which the participant could indicate a symptom not previously mentioned. For data analysis, the following categorization was established: absence of symptoms and persistence of at least one symptom. Long Covid is considered when at least one symptom persists after infection.

To assess resilience levels, the “Resilience Scale” instrument adapted and validated for Brazil was used^(13)^, which aims to measure the levels of positive psychosocial adaptation facing life events. It consists of 25 items described in a positive way, in a Likert-type scale format, with a response range from 1 (completely disagree) to 7 (completely agree). The final score of this scale varies from 25 to 175, the higher the score, the higher the level of resilience. Categorization was performed according to the scores: scores below 125 represent a low level of resilience; from 125 to 145 represents a medium level of resilience; and above 145 a high level of resilience^(14)^. For the inferential analysis of the data, it was dichotomized into: low level of resilience (scores equal to or less than 145); and high level of resilience (scores greater than 145).

Positive mental health was assessed by subjective well-being using the Subjective Well-Being Scale (EBES), adapted and validated for Brazil^(15)^. It is an instrument made up of three subscales: positive affects, negative affects and satisfaction with life. The positive and negative affect subscales are composed of five emotional states each: happy, satisfied, amused, optimistic, and cheerful for the positive affect subscale; and depressed, frustrated, angry, worried, and unhappy for the negative affect subscale. The life satisfaction subscale consists of five statements related to the satisfaction that the individual has with his/her life.

The three subscales are of the Likert type, with responses ranging from 1 to 7 points, where 1 represents not at all and 7 represents extremely. Each of the subscales has a score that varies from 5 to 35 points, and the scores of the items in the negative affect subscale must be inverted to analyze the level of well-being. For this study, only the positive and negative affect subscales were used, and for the analysis the total score of each subscale was used, and the categorization of each one, carried out through the total score value achieved by each individual divided by the number of questions, being considered a high score of subjective well-being (high positive affect and low negative affect) when the final value of this calculation was equal to or greater than four^(15)^.

### Data Collection

Data collection was carried out from July to November 2023, using an online survey form (*Google forms*), through a public invitation was written in the form of an open letter, explaining the research, its objectives, who the participants were (inclusion and exclusion criteria), and the data collection procedures. It was disclosed in specific groups of nursing professionals on social media (*Facebook* and *Instagram*), and messaging app (*Whatsapp*).

After this initial presentation, those interested in participating had to click on the indicated link and were directed to a page with questions regarding the inclusion criteria. When the selection criteria were met, they were directed to the Free Informed Consent Form (FICF), to the acceptance link and, consequently, to the page with the data collection form. If the participant did not meet one of the inclusion criteria or did not agree to participate, or did not accept providing their data for sending the FICF, the form was closed with a thanking message.

### Data Analysis and Treatment

The data was exported from *Google forms* to a spreadsheet *Microsoft Office Mondo 2016 Excel* spreadsheet and, later, analyzed using descriptive and inferential statistics. For descriptive statistics, frequency analyses were carried out.

For the analysis of the relationship between the explanatory variables and the response variable, a Poisson regression model with a robust variance estimator was adjusted and the prevalence ratio was calculated, with a significance level of 5%. This analysis was chosen because it is a technique adjusted for the analysis of binary outcomes in cross-sectional studies, to allow the identification of associated factors and to estimate with greater precision the Prevalence Ratio (PR), which is the measure of association indicated for cross-sectional studies^(16)^.

To adjust the model, the response variable Y was defined with the following coding: Y = 0 for individuals without any symptoms after the acute phase of the disease (*ie*, without long Covid) and Y = 1 for individuals who reported that they remain or have remained with at least one symptom after the acute phase of the disease (*ie*, long Covid). The explanatory variables that entered the model were: age; sex (female/male); professional category (nurse/nursing technician); vaccination against Covid (yes/no); regular practice of physical activity (yes/no); positive affects; negative affects. Missing values (blank responses) in the database were denoted as NA. For continuous variables, missing values (NA) were replaced by the mean of the values recorded. For discrete variables, NA were replaced by the mode of the recorded values.

To adjust and verify the suitability of the model, the data set was first separated into two subsets, called D0 and D1. The D0 data subset comprised 80% of the total dataset. This subset of data was used for the adjusted model performance. In the literature, this type of dataset is called a training dataset. Data subset D1 consisted of the remaining 20% of the data, and was used to verify the performance of the adjusted model.

Since the D0 data set was unbalanced, the SMOTE technique was applied (*Synthetic Minority Oversampling Technique*) to obtain a balanced data set to fit the model, which presented 49.41% of Y = 0 values and 59.59% of Y = 1 values. The likelihood ratio test was applied considering a significance level of a = 0.05 (5%). How the *p*-value was lower than the significance level a considered, the null hypothesis H0 was rejected. In other words, at least one of the explanatory variables is important to explain the response variable Y. To identify which variables are important for the model, the Stepwise method was applied. From this data set, the Poisson regression model with robust variance estimator was adjusted.

### Ethical Aspects

This study was approved by the Human Research Ethics Committee (Opinion number 6.093.840) of the institution to which the research is linked. All participants received information about the study, clarifications about its objectives, data collection procedures, risks and benefits and signed the Free and Informed Consent Form – FICF.

## RESULTS

The study participants were 109 nursing professionals, who were aged from 23 to 68 years old (*mo* = 38), the majority of whom were female (83.5%), nurses (82.6%), who were working in the health area (91.7%), with a monthly salary of two to five minimum wages (49.5%), and were residents in the central-west region (65.1%). It was found that 50 (46.3%) participants responded that they practiced physical activity regularly. Of the 109 participants, 108 (99.1%) were vaccinated against the disease, the majority with three doses or more (Table 1).

**Table 1:** – Freuency of the variables sex, region of residence, professional category, income, vaccination for Covid and regular physical activity among nursing professionals – Campo Grande, MS, Brazil, 2024.

Variables (N)	% (N)
**Sex (N = 109)**	
Female	83.5% (91)
Male	16.5% (18)
**Region in Brazil (N = 109)**	
Northeast	4.6% (5)
Central-West	65.1% (71)
Southeast	20.2% (22)
South	9.2% (10)
North	0.9% (1)
**Professional category (N = 109)**	
Nurse	82.6% (90)
Nursing technician	17.4% (19)
**Approximate income (N = 107)**	
Up to 2 minimum wages	9.3% (10)
Between 2 and 5 minimum wages	49.5% (53)
More than 5 minimum wages	41.1% (44)
**Vaccinated against Covid-19 (N = 109)**	
No	0.9% (1)
Yes	99.1% (108)
**Number of doses of Covid-19 vaccination (N = 108)**	
Didn't get vaccinated	0.9% (1)
1 dose	1.9% (2)
2 doses	7.4% (8)
3 doses	32.4% (35)
4 doses	34.3% (37)
5 doses	23.1% (25)
**Regular physical activity practice (N = 108)**	
No	53.7% (58)
Yes	43.6% (50)
**How many times a week (N = 107)**	
Does not practice activity	54.2% (58)
Once a week	2.8% (3)
2 times a week	4.7% (5)
3 times a week	20.6% (22)
More than 3 times a week	17.8% (19)

%: percentile.

Source: authors’ elaboration.

Regarding the symptoms of Covid-19, 90.8% of professionals stated that they had symptoms that persisted for at least four weeks after the acute phase of the infection and 67.9% reported that symptoms remained/remain after infection (Table 2).

**Table 2:** Freuency of Covid-19 symptoms that remained after the acute phase of the disease in nursing professionals – Campo Grande, MS, Brazil, 2024.

Symptom	Persistence of symptoms for at least 4 weeks after the acute phase of the disease % (N)	Persistence of symptoms % (N)
**Any symptoms present**		
No	9.2% (10)	32.1% (35)
Yes	90.8% (99)	67.9% (74)
**Headache**		
No	64.2% (70)	91.7% (100)
Yes	35.8% (39)	8.3% (9)
**Muscle pain**		
No	72.5% (79)	93.6% (102)
Yes	27.5% (30)	6.4% (7)
**Weakness**		
No	64.2% (70)	95.4% (104)
Yes	35.8% (39)	4.6% (5)
**Cough**		
No	69.7% (76)	96.3% (105)
Yes	30.3% (33)	3.7% (4)
**Shortness of breathe**		
No	78.9% (86)	95.4% (104)
Yes	21.1% (23)	4.6% (5)
**Memory problems/brain fog**		
No	40.4% (44)	56.9% (62)
Yes	59.6% (65)	43.1% (47)
**Sleep disorders**		
No	72.5% (79)	86.2% (94)
Yes	27.5% (30)	13.8% (15)
**Anxiety**		
No	61.5% (67)	78% (85)
Yes	38.5% (42)	22% (24)

%: percentile.

Source: authors’ elaboration.

Other symptoms not mentioned in the questionnaire were also listed by professionals who indicated the persistence of at least one symptom after the acute phase. of Covid-19. Among them, hair loss was reported by six participants, followed by loss of smell by four, tiredness/fatigue by three, loss of taste and dizziness by two. The other symptoms, mentioned by only one participant, were: seizure, insulin resistance, rheumatism, pain, melancholy, asthma, angina, low immunity and sweating.

Regarding mental health variables, most professionals had a low or medium level of resilience and high subjective well-being (high positive affect and low negative affect) (Table 3).

**Table 3:** Freuency of resilience levels and subjective well-being (positive and negative affects) – Campo Grande, MS, Brazil, 2024.

Variable	% (N)
**Resilience Level (N = 104)**	
High	33.7% (35)
Mean	33.7% (35)
Low	32.7% (34)
**Positive affects (N = 107)**	
Low positive affect	27.1% (29)
High positive affect	72.9% (78)
**Negative affects (N = 104)**	
Low negative affect	81.7% (85)
High negative affect	18.3% (19)

%: percentile.

Source: own elaboration.

Regarding sex, more than 70% of female individuals had long Covid. Those participants who had no symptoms for at least four weeks did not have long Covid. More than 90% of participants with low resilience had symptoms of long Covid, as well as those classified as having low positive affect.

Table 4 shows the estimates of the Poisson regression model with robust variance and prevalence ratios, which indicate that four variables are considered significant for explaining long Covid: sex, professional category, resilience and positive affects. The 95% CI results reinforce that the factors are associated as, besides all *p-values* being <0.001, when PR values are less than 1, IC values are too. Similarly, when PR values are greater than 1, IC values are also greater.

**Table 4:** Descriptive analysis of selected variables and Poisson regression analysis with robust variance and prevalence ratio – Campo Grande, MS, Brazil, 2024.

Variable	Long Covid		*p-value*	RP	95% CI
	Yes % (N)	No % (N)			
**Sex**					
Female	73.6% (67)	26.4% (24)	<0.001	1	0.15 to 0.56
Male	38.9% (7)	61.1% (11)		0,29
**Professional Category**				
Nursing technician	73.7% (14)	26.3% (5)	<0.001	1,74	1.27 to 2.37
Nurse	66.7% (60)	33.3% (30)		1	
**Resilience levels**					
Low	91.2% (31)	8.8% (3)	<0.001	1	0.44 to 0.81
High	57.3% (40)	42.7% (30)		0.60	
**Positive affects**					
Low positive affect	96.5% (28)	3.5% (1)	<0.001	1	0.42 to 0.77
High positive affect	57.5% (45)	42.5% (33)		0,57	

p-value: comparison between variables using Poisson regression with robust variance; PR: prevalence ratio; CI: confidence interval; %: percentile.

Source: authors’ elaboration.

Thus, it can be stated that the adjusted prevalence of long Covid in male professionals was 71% lower than the prevalence observed in female professionals. Among nursing technicians, the adjusted prevalence of long Covid was 74% higher when compared to the prevalence of the outcome among nurses. Professionals with high resilience had an adjusted prevalence 40% lower than that among professionals with low resilience. In those with high positive affect, the adjusted prevalence of long COVID was 43% lower compared to the adjusted prevalence in professionals with lower levels of positive affect.

## DISCUSSION

The results of this study allowed identifying that sex, professional category, level of resilience and subjective well-being are predictors of long Covid in nursing professionals.

It is known that the Covid-19 pandemic has brought significant challenges to healthcare professionals, especially those working on the front line, such as nursing staff, which consequently increased the risk of contamination by the Sars-Cov-2 virus. In the present study, most professionals reported the presence of symptoms that persisted after the acute phase of the infection, thus characterizing long Covid. These findings reiterate the greater impact of infection on the physical and mental health, quality of life and functional status of nursing professionals^(1,17)^.

The persistent symptom, after the acute phase of Covid-19, most reported by participants was memory problems/brain fog, which corroborates findings from other studies that indicated a high prevalence of this mental alteration^(18,19)^. The literature also highlights symptoms such as anxiety and depression, sleep disorders and post-traumatic stress disorder^(18)^, and neurological manifestations, such as cognitive disorders, mental confusion, impaired reasoning and attention^(19)^.

Among the participants in the present study, women had a higher prevalence of long Covid. Other studies have also demonstrated this association, with high values such as 84.9%^(20)^ and 81.7^(21)^, which may be related to hormonal and immunological differences between the sexes^(22)^. Thus, biological sex can influence vulnerability to infections, as well as their outcomes, since females have more complete innate and adaptive immune responses, which can prevent an initial infection. However, it makes women more susceptible to autoimmune diseases after the infection has set in^(22)^. Furthermore, another study reveals that the X chromosome present in women can reduce their vulnerability to viral infections, but increases the inflammatory process when infected, which is attributed to the development of long Covid^(23)^.

Regarding the professional category, the nursing technicians participating in this study had a higher prevalence of long Covid in relation to nurses, which may be influenced by the more frequent work of these professionals in carrying out technical procedures and providing direct care to infected individuals. This frequent exposure to the virus increases the risk of contracting the infection and, consequently, developing long Covid. Furthermore, emotional stress, intense workload and adverse conditions can contribute to the emergence of this syndrome in health professionals, especially nursing technicians who sometimes have an increased workload due to the work in more than one job^(1,7)^.

Furthermore, as indicated in another study^(24)^, the incorrect use of Personal Protective Equipment (PPE) appeared as a risk factor for a greater chance of contracting Covid-19 and, consequently, long Covid. Thus, given the different difficulties faced at the beginning of the pandemic, including the inadequate dimensioning of human resources, infrastructure and materials, especially PPE, associated with training for their use, one can reflect on the possible difficulty that the professional category of nursing technicians had in providing safe care^(24)^.

The emotional status of an individual with an acute or chronic condition directly influences the prognosis of the disease. Participants in this study who showed high levels of positive affect had a lower prevalence of long Covid. The study investigating the relationship between positive affects and the recovery of patients with long Covid indicated that those who reported a higher frequency of positive emotions, such as hope and gratitude, showed a faster recovery in symptoms and functionality^(19)^.

Furthermore, increased stress and psychophysical exhaustion at work, both associated with low levels of positive affect, can negatively impact individuals’ mental health and functional status. Research into the mental health of healthcare professionals during the Covid-19 pandemic in Poland found that individuals with higher scores on the stress scale also had high scores on all scales that assess negative aspects of mental health^(3)^.

It is worth noting that the effects of long Covid on mental health can involve several organs and systems, leading to repercussions on the ability to work, carry out daily activities and overall quality of life^(25)^. Thus, high levels of positive affect can constitute an important resource for coping with the impacts caused by long Covid, as evidenced in this study that compared the incidence of long Covid in participants with high levels of positive affect (57.50%) with those with low *levels* of positive affect (96.55%). This result suggests that positive emotional statuses may play a protective role against prolonged complications associated with Covid-19.

Ultimately, resilience is the ability to face adversity, overcome challenges, and adapt to difficult situations. However, individuals with long Covid face a prolonged journey of symptoms and uncertainty^(26)^. The association found in this study between high levels of resilience and lower risk for long Covid is in line with the results of a study also carried out with health professionals, which showed that individuals with increased levels of resilience have a better response to stressors, as well as other positive psychological attributes, proving the advantages of high resilience^(27)^. Another study carried out with individuals with chronic conditions indicated that approximately 57.2% of individuals with low levels of resilience presented significant functional limitations, becoming dependent in their basic daily activities^(28)^.

In turn, a study carried out in Brazil showed that resilient professionals face the symptoms caused by long Covid more easily and are less likely to develop anxiety, stress and depression^(11)^. This is because increased levels of resilience are positively related to life satisfaction, well-being, quality of life, professional achievement, and better coping with adverse situations^(29)^.

The associations identified in this study contribute to the identification of risk groups for long Covid, and emphasize the need to invest in strategies and actions to strengthen the resilience of health professionals and, thus, promote improvements to well-being, increase the ability to face the challenges imposed by the pandemic, contribute to mitigating the impact of long Covid symptoms, and allow both health professionals and individuals in general to face the challenges with greater emotional balance. These strategies need to involve self-care encouragement and support, recognizing each individual’s social network, and seeking appropriate and early treatment^(30)^.

The findings of this study should be interpreted considering its limitations. The first one consists of the sample size that does not represent the entire nursing population. There is also the scope of the responses, due to the restriction on the disclosure of research by certain agencies and sectors, limiting dissemination only to researchers’ public networks. It is worth noting that, despite the limitations, the findings of this research provide valuable insights for strategies to promote well-being, both in the social and professional spheres. These strategies can include training programs, social support, and emotional skills development, broadly benefiting the community.

## CONCLUSION

This study revealed that variables such as sex and professional category are associated with the prevalence of long covid in nursing professionals. Furthermore, high levels of resilience and positive affect are dimensions of mental health that are associated with a lower prevalence of this condition. The identification of these factors, related to long COVID in the studied population, is fundamental for nursing science, which promotes care in all health contexts.

They reinforce the importance of specific care actions for nursing professionals and other health professionals, which involve promotion, prevention, treatment and rehabilitation. To achieve this, multidisciplinary care, centered on the person, and directed at the context and work environment is necessary. Moreover, these findings can support the development of care plans and actions not only for nursing professionals, but also for other individuals who are experiencing this health condition, for quality care.

Additionally, new studies are required to develop interventions that promote the building of resilience and stimulate high positive affects, aiming to mitigate the effects caused by the symptoms of long Covid, and studies on mental health and long Covid that explore other variables such as race and income, for example. This occurs because social and structural determinants of health can explain this relationship or exacerbate it in other contexts and populations.
