# Supplementary material for: Factors associated with long Covid in nursing professionals
Source: Rev Esc Enferm USP. 2025 Jan 31;58:e20240268. doi: 10.1590/1980-220X-REEUSP-2024-0268en (PMC11789625; doi:10.1590/1980-220X-REEUSP-2024-0268en)
Supplement: Translation

# Translation: Fatores associados à Covid longa em profissionais de enfermagem

**DOI:** 10.1590/1980-220X-REEUSP-2024-0268pt

**Authors:** Dayane de Souza Soares Vasconcelos, Daniele Alcalá Pompeo, Mayara Caroline Ribeiro Antonio-Viegas, Adriana Inocenti Miasso, Guilherme Oliveira de Arruda, Elen Ferraz Teston, Erlandson Ferreira Saraiva, Bianca Cristina Ciccone Giacon-Arruda

**Affiliations:** 1 Universidade Federal de Mato Grosso do Sul, Campo Grande, MS, Brasil.; 2 Faculdade de Medicina de São José do Rio Preto, São José do Rio Preto, SP, Brasil.; 3 Universidade Federal de Mato Grosso do Sul, Três Lagoas, MS, Brasil.; 4 Universidade de São Paulo, Escola de Enfermagem de Ribeirão Preto, Ribeirão Preto, SP, Brasil.

**Keywords:** Síndrome Pós-COVID-19 Aguda, Profissionais de Enfermagem, Resiliência psicológica, Saúde Mental

## Abstract

**Objetivo:**

Analisar a associação entre variáveis sociodemográficas, hábitos de vida e de saúde mental e a Covid longa em profissionais de enfermagem.

**Método:**

Estudo quantitativo, observacional, transversal e analítico, com 109 profissionais de enfermagem que tiveram Covid-19 entre os anos de 2020 e 2022. A coleta de dados foi por formulário online, com as variáveis de interesse: resiliência, bem-estar subjetivo, idade, sexo, categoria profissional, vacinação, atividade física, presença de sintomas decorrentes da infecção pela Covid-19 após a fase aguda da doença e de Covid longa. Os dados foram analisados de maneira descritiva, e inferencial pela regressão de Poisson com variância robusta, e nível de significância de 5%.

**Resultados:**

Sexo masculino, alta resiliência e alto afeto positivo diminui a prevalência da Covid longa em 71% (RP = 0,29), 40% (RP = 0,60) e 43% (RP = 0,57), respectivamente, enquanto ser da categoria técnico de enfermagem aumenta em 74% (RP = 1,74) a sua prevalência.

**Conclusión:**

Os resultados reforçam a importância e subsidiam o desenvolvimento de ações de promoção, prevenção, tratamento e reabilitação aos indivíduos com Covid longa. Para isso, o cuidado multidisciplinar, centrado na pessoa e direcionado ao contexto e ambiente de trabalho se fazem necessários.

## INTRODUÇÃO

No período de 2019 a 2023, o mundo foi acometido pela pandemia de Covid-19, e vivenciou um estado de emergência em saúde em nível mundial^(1)^. Em 2020, aproximadamente 90 mil profissionais de saúde foram infectados em 30 países^(1)^. No Brasil, até junho de 2023, os profissionais de enfermagem infectados pelo vírus da Covid-19 somavam um total de 65.029, sendo 872 o número de óbitos, resultando em uma letalidade entre a classe de 2,27%^(2)^.

Após o controle e fim do estado pandêmico, potencializado principalmente pela vacinação, estudos têm investigado uma condição de saúde pós infecção viral e descrito as diferentes alterações permanentes na saúde física e mental das pessoas que tiveram a doença^(3,4)^ denominada Covid longa^(5)^. Esta condição pode surgir até três meses após a fase aguda da infecção, com duração de, pelo menos, dois meses^(5)^.

Estima-se, em nível mundial, que 200 milhões de indivíduos tenham tido sintomas prolongados pós infecção aguda pelo vírus da Covid-19, com uma prevalência de 43% dos que foram infectados^(6)^. No Brasil, um estudo realizado com 646 indivíduos^(7)^ mostrou que cerca de 50,2% desenvolveram essa condição. Embora ela possa afetar qualquer pessoa, mulheres e profissionais de saúde parecem estar em maior risco^(8)^.

A Covid longa apresenta-se de diferentes formas nos indivíduos e pode envolver um ou mais sintomas, como fadiga, perda do olfato ou paladar, persistência de tosse, dor de cabeça, dores musculares e articulares, fraqueza e mal-estar. Além disso, comprometimento das funções psicomotoras, alterações neuropsíquicas como dificuldade de concentração e perda da memória ou nevoeiro cerebral, tontura, declínio da habilidade de linguagem e fluência verbal são observados^(9)^.

Estes sintomas impactam a capacidade das pessoas de retornar às atividades de vida diárias, laborais e sociais, o que gera consequências econômicas e para a saúde mental desses indivíduos^(5,8)^. Os estudos disponíveis concentram-se na investigação de sinais e sintomas da Covid longa^(7)^, na vivência da pandemia, e seus efeitos na saúde dos profissionais^(10)^, e na influência positiva da resiliência apresentada por profissionais de saúde no enfrentamento dos sintomas causados pela Covid longa^(11)^. Desse modo, torna-se premente a investigação de fatores associados à Covid longa, a fim de contribuir para o planejamento de ações estratégicas de cuidado para os profissionais de saúde, em especial no que tange aos hábitos de vida e de saúde mental.

Frente ao exposto, este estudo teve como objetivo analisar a associação entre variáveis sociodemográficas, hábitos de vida e de saúde mental e a Covid longa em profissionais de enfermagem.

## MÉTODO

### Desenho do Estudo

Trata-se de estudo de abordagem quantitativa, do tipo observacional, transversal e analítico. Este estudo deriva de um estudo matriz, com multivariáveis denominado “A Covid longa em profissionais de enfermagem e suas implicações na saúde mental e no estado funcional”. Para sua estrutura e organização, seguiram-se as recomendações da declaração *Strengthening the Reporting of Observational Studies in Epidemiology* (STROBE)^(12)^.

### Local e População do Estudo

O estudo teve abrangência nacional, com profissionais de enfermagem (técnico de enfermagem e enfermeiro) residentes em qualquer localidade do Brasil. Os critérios de inclusão foram: ser profissional de enfermagem, de ambos os sexos, que referiram ter testado positivo para Covid-19 por meio do teste RT-PCR, no período de 2020 a 2022, independente da gravidade e das medidas de tratamento realizadas. Foram excluídos aqueles que referiram não ter realizado o teste de Covid-19 ou que o teste realizado não foi o teste RT-PCR.

A amostra do estudo foi não probabilística, por conveniência, e consistiu em 109 profissionais de enfermagem (técnico de enfermagem e enfermeiro).

### Variáveis de Interesse e Instrumentos de Coleta de Dados

As variáveis de interesse desse estudo foram definidas como: 1) variável resposta - Covid longa; 2) variáveis explicativas – níveis de resiliência, bem-estar subjetivo (afetos positivos e negativos), idade, sexo, categoria profissional, vacinação para Covid (sim/não), prática de atividade física (sim/não) e presença de sintomas decorrentes da infecção pela Covid-19, após a fase aguda da doença.

Para as variáveis idade, sexo (feminino/masculino), categoria profissional (enfermeiro(a)/técnico(a) de enfermagem), vacinação para Covid (sim/não) e prática de atividade física (sim/não) foi construído um instrumento pelos pesquisadores do estudo que continha, além dessas variáveis, outras como: município e estado de moradia, renda, local de trabalho atual e quando testou positivo para Covid, se atua na área atualmente, medicações de uso contínuo e diagnóstico de outras condições de saúde.

As variáveis sintomas de Covid após a infecção aguda e Covid longa foram avaliadas por meio de um questionário próprio, também desenvolvido pelos pesquisadores desse estudo, baseado em um estudo prévio que analisou os impactos da pandemia na saúde de enfermeiros envolvendo os principais sintomas prolongados identificados na literatura^(4)^: dor de cabeça; dores musculares; fraqueza; tosse; falta de ar; problemas de memória/nevoeiro cerebral; distúrbios do sono; e ansiedade. Para esses sintomas, foi solicitado para o participante indicar: 1) quais persistiram, por no mínimo, quatro semanas após a fase aguda; e 2) quais sintomas permaneceram/permanecem.

Ademais, o questionário continha uma questão em que o participante poderia indicar algum sintoma não mencionado anteriormente. Para a análise de dados, estabeleceu-se a seguinte categorização: ausência de sintomas e permanência de pelo menos um sintoma. Foi considerada Covid longa a permanência de pelo menos um sintoma após a infecção.

Para a avaliação dos níveis de resiliência, utilizou-se o instrumento “Escala de resiliência” adaptado e validado para o Brasil^(13)^, que tem como objetivo mensurar os níveis de adaptação psicossociais positivas frente a eventos da vida. Ela é constituída por 25 itens descritos de forma positiva, em formato de escala do tipo likert, com variação de resposta de 1 (discordo totalmente) a 7 (concordo totalmente). O escore final desta escala varia de 25 a 175, e quanto maior o escore, maior o nível de resiliência. A categorização foi realizada de acordo com os escores: escoresabaixo de 125 representam baixo nível de resiliência; de 125 a 145 representam nível médio de resiliência; e acima de 145 um alto nível de resiliência^(14)^. Para a análise inferencial dos dados, dicotomizou-se em: baixo nível de resiliência (escores iguais ou menores que 145); e alto nível de resiliência (escores acima de 145).

A saúde mental positiva foi avaliada pelo bem-estar subjetivo por meio da Escala de Bem-estar Subjetivo (EBES), adaptada e validada para o Brasil^(15)^. É um instrumento formado por três subescalas: afetos positivos, afetos negativos e satisfação com a vida. As subescalas de afetos positivos e negativos são compostas por cinco estados emocionais cada: feliz, satisfeito, divertido, otimista e alegre para a subescala de afeto positivo; e deprimido, frustrado, raivoso, preocupado e infeliz para a de afetos negativos. A subescala de satisfação com a vida é composta de cinco afirmações relacionadas à satisfação que o indivíduo tem com sua vida.

As três subescalas são do tipo likert, com respostas que variam de 1 a 7 pontos, em que 1 representa nada e 7 extremamente. Cada uma das subescalas tem o score que varia de 5 a 35 pontos, sendo que a pontuação dos itens da subescala de afetos negativos tem que ser invertida, para analisar o nível de bem-estar. Para este estudo, utilizaram-se apenas as subescalas de afetos positivos e negativos, e para a análise foi utilizado o escore total de cada subescala, e a categorização de cada uma, realizada por meio do valor total de escore alcançado por cada indivíduo dividido pelo número de questões, sendo considerado escore alto de bem-estar subjetivo (alto afeto positivo e baixo afeto negativo) quando o valor final desse cálculo foi igual ou maior que quatro^(15)^.

### Coleta de Dados

A coleta de dados foi realizada no período de julho a novembro de 2023, com formulário da pesquisa online (*google forms*), por meio de um convite público redigido em forma de carta aberta, explicando sobre a pesquisa, seus objetivos, quem eram os participantes (critérios de inclusão e exclusão) e os procedimentos de coleta de dados. Ele foi divulgado em grupos específicos de profissionais de enfermagem nas redes sociais (*facebook* e *instagram*), e aplicativo de mensagem (*whatsapp*).

Após essa apresentação inicial, os interessados em participar deveriam clicar no link indicado e eram direcionados a uma página com as questões referentes aos critérios de inclusão. Quando preenchidos os critérios de seleção, eram direcionados ao Termo de Consentimento Livre e Esclarecido (TCLE), ao link de aceite e, conseguinte, para a página com o formulário de coleta de dados. No caso em que o participante não atendesse a um dos critérios de inclusão ou não concordasse em participar, ou não aceitasse informar seus dados para envio do TCLE, o formulário era encerrado com uma mensagem de agradecimento.

### Análise e Tratamento dos dados

Os dados foram exportados do *google forms* para uma planilha do *Excel do Microsoft Office Mondo 2016 e*, posteriormente, analisados por meio de estatística descritiva e inferencial. Para a estatística descritiva foram realizadas análises de frequência.

Para a análise da relação entre as variáveis explicativas e a variável resposta, ajustou-se um modelo de regressão de Poisson com estimador de variância robusta e calculou-se a razão de prevalência, e nível de significância de 5%. Optou-se por essa análise por se tratar de técnica ajustada para análise de desfechos binários em estudos transversais, de modo a possibilitar a identificação de fatores associados e estimar com maior precisão a Razão de Prevalência (RP), que se trata da medida de associação indicada para estudos transversais^(16)^.

Para o ajuste do modelo, definiu-se a variável resposta Y com a seguinte codificação: Y = 0 para indivíduos sem nenhum sintoma após a fase aguda da doença (*i.e.*, sem covid longa) e Y = 1 para indivíduos que informaram que permanecem ou tenham permanecido com pelo menos um sintoma após a fase aguda da doença (*i.e.*, covid longa). As variáveis explicativas que entraram no modelo foram: idade; sexo (feminino/masculino); categoria profissional (enfermeiro/técnico de enfermagem); vacinação para covid (sim/não); prática regular de atividade física (sim/não); afetos positivos; afetos negativos. Os valores faltantes (respostas em branco) na base de dados foram denotados como NA. Para as variáveis contínuas, os valores faltantes (NA) foram substituídos pela média dos valores registrados. E para as variáveis discretas, os NA foram substituídos pela moda dos valores registrados.

Para o ajuste e verificação da adequabilidade do modelo, primeiramente, separou-se o conjunto de dados em dois subconjuntos, denominados de D0 e D1. O subconjunto de dados D0 foi composto por 80% da base de dados do conjunto total. Este subconjunto de dados foi utilizado para o ajuste do modelo. Na literatura, este tipo de conjunto de dados é denominado de conjunto de dados de treinamento. O subconjunto de dados D1 foi composto pelos 20% dos dados restantes, e foi utilizado para verificar a performance do modelo ajustado.

Como o conjunto de dados D0 apresentava um desbalanceamento, aplicou-se a técnica SMOTE (*Synthetic Minority Oversampling Technique*) para obter um conjunto de dados balanceado para ajustar o modelo, que apresentou 49,41% de valores Y = 0 e 59,59% de valores Y = 1. O teste da razão de verossimilhanças foi aplicado considerando um nível de significância a = 0,05 (5%). Como o *p*-valor foi menor do que o nível de significância a considerado, a hipótese nula H0 foi rejeitada. Ou seja, pelo menos uma das variáveis explicativas é importante para explicar a variável resposta Y. Para identificar quais variáveis são importantes para o modelo, aplicou-se o método Stepwise. A partir desse conjunto de dados, o modelo de regressão de Poisson com estimador de variância robusta foi ajustado.

### Aspectos Éticos

O presente estudo foi aprovado pelo Comitê de Ética em Pesquisa Envolvendo Seres Humanos (Parecer número 6.093.840) da instituição à qual a pesquisa está vinculada. Todos os participantes receberam informações sobre o estudo, esclarecimentos sobre seus objetivos, procedimentos de coleta de dados, riscos e benefícios e assinaram o Termo de Consentimento Livre e Esclarecido – TCLE.

## RESULTADOS

Os participantes do estudo foram 109 profissionais de enfermagem, que tinham idade de 23 a 68 anos (*mo* = 38), sendo a maioria do sexo feminino (83,5%), enfermeiros (82,6%), que estavam atuando na área da saúde (91,7%), com remuneração mensal de dois a cinco salários-mínimos (49,5%), e residentes na região centro-oeste (65,1%). Verificou-se que 50 (46,3%) participantes responderam que praticavam atividade física regularmente. Dos 109 participantes, 108 (99,1%) vacinaram-se contra a doença, a maioria com três doses ou mais (Tabela 1).

**Tabela 1:** Freuência das variáveis sexo, região de residencia, categoria profissional, renda, vacinação para Covid e prática de atividade física regular em profissionais de enfermagem – Campo Grande, MS, Brasil, 2024.

Variáveis (N)	% (N)
**Sexo (N = 109)**	
Feminino	83,5% (91)
Masculino	16,5% (18)
**Região Brasil (N = 109)**	
Nordeste	4,6% (5)
Centro Oeste	65,1% (71)
Sudeste	20,2% (22)
Sul	9,2% (10)
Norte	0,9% (1)
**Categoria profissional (N = 109)**	
Enfermeiro	82,6% (90)
Técnico em enfermagem	17,4% (19)
**Renda aproximada (N = 107)**	
Até 2 salários-mínimos	9,3% (10)
Entre 2 e 5 salários-mínimos	49,5% (53)
Mais ue 5 salários-mínimos	41,1% (44)
**Vacinou-se contra a Covid-19 (N = 109)**	
Não	0,9% (1)
Sim	99,1% (108)
**Número de doses da vacinação contra Covid-19 (N = 108)**	
Não se vacinou	0,9% (1)
1 dose	1,9% (2)
2 doses	7,4% (8)
3 doses	32,4% (35)
4 doses	34,3% (37)
5 doses	23,1% (25)
**Prática de atividade física regular (N = 108)**	
Não	53,7% (58)
Sim	43,6% (50)
uantas vezes por semana (N = 107)	
Não pratica atividade	54,2% (58)
1 vez por semana	2,8% (3)
2 vezes por semana	4,7% (5)
3 vezes por semana	20,6% (22)
Mais ue 3 vezes por semana	17,8% (19)

%: percentil.

Fonte: elaboração própria.

Em relação aos sintomas da Covid-19, 90,8% dos profissionais afirmaram que tiveram sintomas que persistiram por, no mínimo, quatro semanas após a fase aguda da infecção e 67,9% informaram que os sintomas permaneceram/permanecem após a infecção (Tabela 2).

**Tabela 2:** Freuência dos sintomas da Covid-19 ue permaneceram após a fase aguda da doença em profissionais de enfermagem – Campo Grande, MS, Brasil, 2024.

Sintoma	Permanência de sintomas por, no mínimo, 4 semanas após a fase aguda da doença % (N)	Permanência de sintomas % (N)
**Qualquer sintoma presente**		
Não	9,2% (10)	32,1% (35)
Sim	90,8% (99)	67,9% (74)
**Dor de cabeça**		
Não	64,2% (70)	91,7% (100)
Sim	35,8% (39)	8,3% (9)
**Dores musculares**		
Não	72.5 % (79)	93,6% (102)
Sim	27.5 % (30)	6,4% (7)
**Fraueza**		
Não	64.2 % (70)	95,4% (104)
Sim	35.8 % (39)	4,6% (5)
**Tosse**		
Não	69,7% (76)	96,3% (105)
Sim	30,3% (33)	3,7% (4)
**Falta de ar**		
Não	78,9% (86)	95,4% (104)
Sim	21,1% (23)	4,6% (5)
**Problemas de memória/nevoeiro cerebral**		
Não	40,4% (44)	56,9% (62)
Sim	59,6% (65)	43,1% (47)
**Distúrbios do sono**		
Não	72,5% (79)	86,2% (94)
Sim	27,5% (30)	13,8% (15)
**Ansiedade**		
Não	61,5% (67)	78% (85)
Sim	38,5% (42)	22% (24)

%: percentil.

Fonte: elaboração própria.

Outros sintomas não citados no questionário também foram listados pelos profissionais que indicaram a permanência de pelo menos um sintoma após a fase aguda da Covid-19. Dentre eles, a queda de cabelo foi indicada por seis participantes, seguida pela perda de olfato por quatro, cansaço/fadiga por três, perda de paladar e tontura por dois. Os demais sintomas, citados apenas por um participante, foram: convulsão, resistência à insulina, reumatismo, dores, melancolia, asma, angina, baixa imunidade e sudorese.

Em relação às variáveis de saúde mental, a maioria dos profissionais tinha baixo ou médio nível de resiliência e alto bem-estar subjetivo (alto afeto positivo e baixo afeto negativo) (Tabela 3).

**Tabela 3:** Freuência dos níveis de resiliência e de bem-estar subjetivo (afetos positivos e negativos) – Campo Grande, MS, Brasil, 2024.

Variável	% (N)
**Nível de Resiliência (N=104)**	
Alta	33,7% (35)
Média	33,7% (35)
Baixa	32,7% (34)
**Afetos positivos (N=107)**	
Baixo afeto positivo	27,1% (29)
Alto afeto positivo	72,9% (78)
**Afetos negativos (N=104)**	
Baixo afeto negativo	81,7% (85)
Alto afeto negativo	18,3% (19)

%: percentil.

Fonte: elaboração própria.

Em relação ao sexo, mais de 70% dos indivíduos do sexo feminino apresentaram Covid longa. Aqueles participantes que não tiveram sintomas por, no mínimo, quatro semanas, não tiveram Covid longa. Mais de 90% dos participantes com baixa resiliência apresentaram sintomas de Covid longa, assim como aqueles classificados com baixo afeto positivo.

Na Tabela 4 há as estimativas do modelo de regressão de Poisson com variância robusta e razões de prevalência, que indicam que quatro variáveis são consideradas significativas para explicação da Covid longa: sexo, categoria profissional, resiliência e afetos positivos. Os resultados de IC٩٥٪ reforçam que os fatores são associados pois, além de todos os *p-valores* serem <0,001, quando os valores de RP são menores do que 1, os valores de IC também são. De maneira semelhante, quando os valores da RP são maiores do que 1, os valores de IC também são maiores.

**Tabela 4:** Análise descritiva das variáveis selecionadas e análise de regressão de Poisson com variância robusta e razão de prevalência – Campo Grande, MS, Brasil, 2024.

Variável	Covid Longa		*p-valor*	RP	I.C 95%
	Sim % (N)	Não % (N)			
**Sexo**					
Feminino	73,6% (67)	26,4% (24)	<0,001	1	0,15 a 0,56
Masculino	38,9% (7)	61,1% (11)		0,29	
**Categoria Profissional**					
Técnico de enfermagem	73,7% (14)	26,3% (5)	<0,001	1,74	1,27 a 2,37
Enfermeiro	66,7% (60)	33,3% (30)		1	
**Níveis de resiliência**					
Baixo	91,2% (31)	8,8% (3)	<0,001	1	0,44 a 0,81
Alto	57,3% (40)	42,7% (30)		0,60	
**Afetos positivos**					
Baixo afeto positivo	96,5% (28)	3,5% (1)	<0,001	1	0,42 a 0,77
Alto afeto positivo	57,5% (45)	42,5% (33)		0,57	

p-valor: comparação entre as variáveis por meio da regressão de Poisson com variância robusta; RP: razão de prevalência; I.C: intervalo de confiança; %: percentil.

Fonte: elaboração própria.

Assim, pode-se afirmar que a prevalência ajustada de covid longa em profissionais do sexo masculino foi 71% menor do que a prevalência observada em profissionais do sexo feminino. Entre técnicos de enfermagem, a prevalência ajustada de covid longa foi 74% maior, quando comparada à prevalência do desfecho entre enfermeiros. Os profissionais com alta resiliência apresentaram prevalência ajustada 40% menor do que entre os profissionais com baixa resiliência. Naqueles com alto afeto positivo, a prevalência ajustada de covid longa foi 43% menor em relação prevalência ajustada nos profissionais com níveis mais baixos de afeto positivo.

## DISCUSSÃO

Os resultados deste estudo permitiram identificar que o sexo, a categoria profissional, o nível de resiliência e o bem- estar subjetivo são preditores da Covid longa em profissionais de enfermagem.

Sabe-se que a pandemia da Covid-19 trouxe desafios significativos para os profissionais de saúde, especialmente para aqueles que atuam na linha de frente, como por exemplo a equipe de enfermagem, o que, consequentemente, aumentou o risco de contaminação pelo vírus Sars-Cov-2. No presente estudo, a maioria dos profissionais referiu a presença de sintomas que persistiram após a fase aguda da infecção, caracterizando, assim, a Covid longa. Estes achados reiteram o maior impacto da infecção na saúde física, mental, qualidade de vida e estado funcional dos profissionais de enfermagem^(1,17)^.

O sintoma persistente, após a fase aguda da Covid-19, mais relatado pelos participantes foi problemas de memória/nevoeiro cerebral, o que corrobora achados de outros estudos que indicaram alta prevalência dessa alteração mental^(18,19)^. A literatura também evidencia sintomas como ansiedade e depressão, distúrbios do sono e estado pós-traumático^(18)^, e manifestações neurológicas, como distúrbios cognitivos, confusão mental, comprometimento do raciocínio e da atenção^(19)^.

Dentre os participantes do presente estudo, as mulheres apresentaram maior prevalência de Covid longa. Outros estudos também demonstraram essa associação, com valores elevados como 84,9%^(20)^ e 81,7%^(21)^, o que pode estar relacionado às diferenças hormonais e imunológicas entre os sexos^(22)^. Desse modo, o sexo biológico pode influenciar a vulnerabilidade para infecções, assim como para seus desfechos, visto que o sexo feminino possui respostas imunes inatas e adaptativas mais completas, o que pode impedir uma infecção inicial. Entretanto, torna as mulheres mais suscetíveis a doenças autoimunes após a infecção instalada^(22)^. Ademais, outro estudo revela que o cromossomo X presente em mulheres pode reduzir sua vulnerabilidade para infecções virais, porém aumenta o processo inflamatório quando infectadas, o que é atribuído ao desenvolvimento da Covid longa^(23)^.

Referente categoria profissional, os técnicos de enfermagem participantes do presente estudo apresentaram maior prevalência de Covid longa em relação aos enfermeiros, o que pode ter influência da atuação mais frequente desses profissionais na realização de procedimentos técnicos e de cuidados diretos aos indivíduos infectados. Essa exposição frequente ao vírus aumenta o risco de contrair a infecção e, consequentemente, de desenvolver a Covid longa. Além disso, o estresse emocional, a carga de trabalho intensa e condições adversas podem contribuir para o surgimento dessa síndrome em profissionais de saúde, sobretudo os técnicos em enfermagem que, por vezes, têm uma carga horária aumentada por possuírem mais de um vínculo empregatício^(1,7)^.

Ademais, como indicado em outro estudo^(24)^, o uso incorreto dos Equipamentos de Proteção Individual (EPI’s) apareceu como um fator de risco para maior chance de contrair Covid-19 e, consequentemente, Covid longa. Assim, frente às diferentes dificuldades enfrentadas no início da pandemia, dentre elas, o dimensionamento inadequado de recursos humanos, de infraestrutura e materiais, principalmente de EPIs, associadas à capacitação para o seu uso, pode-se refletir sobre a possível dificuldade que a categoria profissional dos técnicos em enfermagem possuiu para a oferta de cuidado seguro^(24)^.

O estado emocional do indivíduo com uma condição aguda ou crônica influencia diretamente o prognóstico da doença. Os participantes deste estudo que apresentaram níveis elevados de afeto positivo tiveram menor prevalência de Covid longa. Estudo que investigou a relação entre afetos positivos e a recuperação de pacientes com Covid longa indicou que aqueles que relataram maior frequência de emoções positivas, como esperança e gratidão, apresentaram uma recuperação mais rápida nos sintomas e na funcionalidade^(19)^.

Além disso, o aumento do estresse e o esgotamento psicofísico no trabalho, ambos associados a baixos níveis de afetos positivos, podem impactar negativamente a saúde mental e o estado funcional dos indivíduos. Pesquisa sobre a saúde mental dos profissionais de saúde durante a pandemia da Covid-19 na Polônia apontou que indivíduos com pontuações mais altas na escala de estresse também apresentaram pontuações elevadas em todas as escalas que avaliam aspectos negativos da saúde mental^(3)^.

Cabe salientar que os efeitos da Covid longa na saúde mental podem envolver diversos órgãos e sistemas, causando repercussões na capacidade de trabalhar, realizar atividades diárias e na qualidade de vida em geral^(25)^. Assim, níveis elevados de afetos positivos podem constituir recurso importante para o enfrentamento dos impactos ocasionados pela Covid longa, como evidenciado neste estudo que comparou a incidência de Covid longa em participantes com níveis altos de afeto positivo (57,50%) com aqueles com *níveis* baixos de afeto positivo (96,55%). Este resultado sugere que estados emocionais positivos podem desempenhar um papel protetor contra as complicações prolongadas associadas à Covid-19.

Por fim, a resiliência é a capacidade de enfrentar adversidades, superar desafios e se adaptar a situações difíceis, todavia, indivíduos com Covid longa enfrentam uma jornada prolongada de sintomas e incertezas^(26)^. A associação verificada neste estudo entre níveis elevados de resiliência e menor risco para Covid longa coaduna com resultados de uma investigação realizada também com profissionais de saúde, a qual apontou que indivíduos com nível de resiliência aumentado apresentam melhor resposta a agentes estressores, assim como demais atributos psicológicos positivos, comprovando as vantagens de uma alta resiliência^(27)^. Outro estudo realizado com indivíduos com condições crônicas indicou que cerca de 57,2% dos indivíduos com baixo nível de resiliência apresentaram limitações funcionais significativas, tornando-se dependentes em suas atividades básicas diárias^(28)^.

Por sua vez, estudo realizado no Brasil apontou que profissionais resilientes enfrentam os sintomas causados pela Covid longa com mais facilidade e são menos predispostos a desenvolver ansiedade, estresse e depressão^(11)^. Isso porque os níveis aumentados de resiliência estão relacionados de forma positiva com satisfação com a vida, bem-estar, qualidade de vida, realização profissional e melhor enfrentamento a situações adversas^(29)^.

As associações identificadas nesse estudo contribuem para a identificação de grupos de risco para a Covid longa, e reforçam a necessidade de investir em estratégias e ações para fortalecer a resiliência de profissionais de saúde e, com isso, promover melhorias no bem-estar, aumentar a capacidade de enfrentar os desafios impostos pela pandemia, contribuir para mitigar o impacto dos sintomas da Covid longa e permitir que tanto os profissionais da saúde como indivíduos em geral enfrentem os desafios com maior equilíbrio emocional. Essas estratégias necessitam envolver o incentivo e apoio ao autocuidado, o reconhecimento da rede social de cada indivíduo e a busca por tratamento adequado e precoce^(30)^.

Os achados deste estudo devem ser interpretados considerando suas limitações. A primeira consiste no tamanho da amostra que não representa toda a população da enfermagem. Tem-se, ainda, o alcance das respostas devido à restrição de divulgação da pesquisa por determinados órgãos e setores, limitando-se a divulgação apenas nas redes públicas dos pesquisadores. Destaca-se que, apesar das limitações, os achados desta pesquisa fornecem insights valiosos para estratégias de promoção do bem-estar, tanto no âmbito social quanto profissional. Essas estratégias podem incluir programas de treinamento, apoio social e desenvolvimento de habilidades emocionais, beneficiando amplamente a comunidade.

## CONCLUSÃO

Este estudo revelou que variáveis como sexo e categoria profissional estão associadas com a prevalência de covid longa em profissionais de enfermagem. Além disso, altos níveis de resiliência e afetos positivos são dimensões da saúde mental que estão associadas a uma menor prevalência dessa condição. A identificação desses fatores, relacionados à covid longa na população estudada, é fundamental para a ciência da enfermagem, que promove o cuidado em todos os contextos da saúde.

Eles reforçam a importância de ações de cuidados específicas aos profissionais de enfermagem, e outros profissionais de saúde, que envolvam a promoção, a prevenção, o tratamento e a reabilitação. Para isso, o cuidado multidisciplinar, centrado na pessoa, e direcionados ao contexto e ambiente de trabalho se fazem necessários. Ademais, esses achados podem subsidiar o desenvolvimento de planos e ações de cuidado não apenas aos profissionais de enfermagem, mas se estender a outros indivíduos que estejam vivenciando essa condição de saúde, para uma assistência de qualidade.

Adicionalmente, são necessários novos estudos para desenvolver intervenções que promovam a construção de resiliência e estimulem altos afetos positivos, visando mitigar os efeitos causados pelos sintomas da Covid longa, e estudos sobre a saúde mental e a Covid longa que explorem outras variáveis como a cor e a renda, por exemplo. Isso porque os determinantes sociais e estruturais de saúde podem explicar essa relação ou exacerbá-la em outros contextos e populações.
